# The system of care for the diabetic foot: objectives, outcomes, and opportunities

**DOI:** 10.3402/dfa.v4i0.21847

**Published:** 2013-10-10

**Authors:** Neal R. Barshes, Meena Sigireddi, James S. Wrobel, Archana Mahankali, Jeffrey M. Robbins, Panos Kougias, David G. Armstrong

**Affiliations:** 1 Division of Vascular Surgery and Endovascular Therapy, Michael E. DeBakey Department of Surgery, Baylor College of Medicine/Michael E. DeBakey Veterans Affairs Medical Center, Houston, TX, USA; 2 Paul L. Foster School of Medicine, Texas Tech University Health Sciences Center, El Paso, TX, USA; 3 Division of Metabolism, Endocrinology and Diabetes, Department of Internal Medicine, University of Michigan Medical School, Ann Arbor, MI, USA; 4 Primary Care Line, Michael E. DeBakey Veterans Affairs Medical Center, Houston, TX, USA; 5 Louis Stokes VA Medical Center, Cleveland, OH, USA; 6 Department of Surgery, Southern Arizona Limb Salvage Alliance (SALSA), University of Arizona College of Medicine, Tucson, AZ, USA

**Keywords:** foot ulcer, diabetes, peripheral vascular disease, diabetic neuropathy, delivery of healthcare, physician's practice patterns

## Abstract

Most cases of lower extremity limb loss in the United States occur among people with diabetes who have a diabetic foot ulcer (DFU). These DFUs and the associated limb loss that may occur lead to excess healthcare costs and have a large negative impact on mobility, psychosocial well-being, and quality of life. The strategies for DFU prevention and management are evolving, but the implementation of these prevention and management strategies remains challenging. Barriers to implementation include poor access to primary medical care; patient beliefs and lack of adherence to medical advice; delays in DFU recognition; limited healthcare resources and practice heterogeneity of specialists. Herein, we review the contemporary outcomes of DFU prevention and management to provide a framework for prioritizing quality improvement efforts within a resource-limited healthcare environment.

Approximately 84% of non-traumatic major amputations among people with diabetes are preceded by a diabetic foot ulcer (DFU) (1). These DFUs – defined as any necrosis, gangrene, or full-thickness skin defect occurring distal to the ankle in a diabetic patient (2) – serve as the portal of entry for severe foot infections, and the end-stage complication may be limb loss through major (above-ankle) amputation (Fig. 1). DFUs are analogous to many cancers in that the diagnosis and management of certain identifiable/visible precursor states may halt progression of disease and reduce end-stage complications (3) (Table 1).

**Fig. 1:**
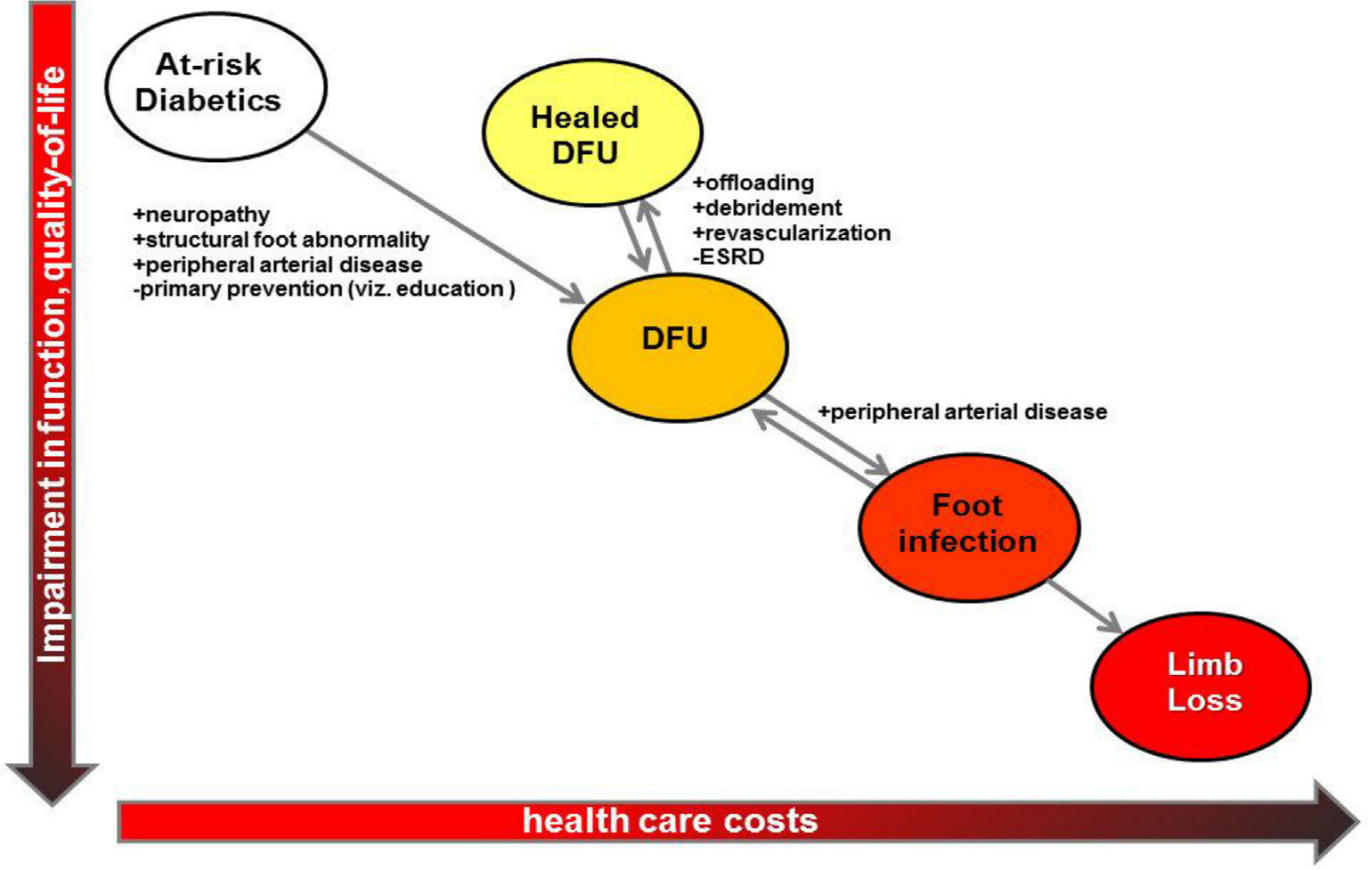
The clinical states leading to limb loss among patients with diabetes mellitus and the risk factors that influence the transition between these states. DFU=diabetic foot ulcer.

**Table 1:** A comparison of the burden of disease, detection, and management of colorectal cancer and diabetes-associated limb loss

Characteristic	Colorectal cancer	Diabetic limb loss
Precursor	Colorectal polyp	Diabetic foot ulcer
Screening modality	Colonoscopy, double-contrast barium enema	Annual foot examination for diabetic patients
Occurrence of precursor	21% incidence at age 50	15%
Other diagnostic modalities	Computed tomography of abdomen and pelvis; whole-body positron emission tomography (PET) scan	Plain foot X-rays; MRI; positron emission tomography-computed tomography (PET-CT); tagged white blood cell (WBC) scan
Incidence of disease	5.1% lifetime incidence (4)	15% lifetime incidence among people with diabetes
	46/100K population (4)	130/100K persons with diabetes
Annual number of cases of disease	143,000 in 2012 (4)	34,000 in 2009
Specialties involved in management	Gastroenterologists; general and colorectal surgeons	Podiatrists; vascular surgeons; general surgeons; orthopedic surgeons; infectious disease specialists; endocrinologists; prosthetists/orthotists
Estimated US annual costs (in US dollars)	14 billion (5)	17 billion

Multiple large-scale studies of patient self-reported quality of life have shown that limb loss has a larger negative impact on quality of life than any other complication of diabetes, including end-stage renal disease or blindness (6, 7). In addition to the loss of mobility and independence (8, 9), depression and anxiety are very prevalent among people with diabetes who have experienced limb loss (10–12). The economic costs associated with diabetic foot care – including amputation care – represent the single largest category of excess medical costs associated with diabetes (13). The total cost for diabetic foot care for those with neuropathy has been estimated to be $11 billion (14). Even conservatively extrapolating these figures to include those with diabetes and peripheral arterial disease (PAD) would increase the total cost estimate to $17 billion, comparable to the annual costs of breast cancer and colorectal cancer (5) (Fig. 2).

**Fig. 2:**
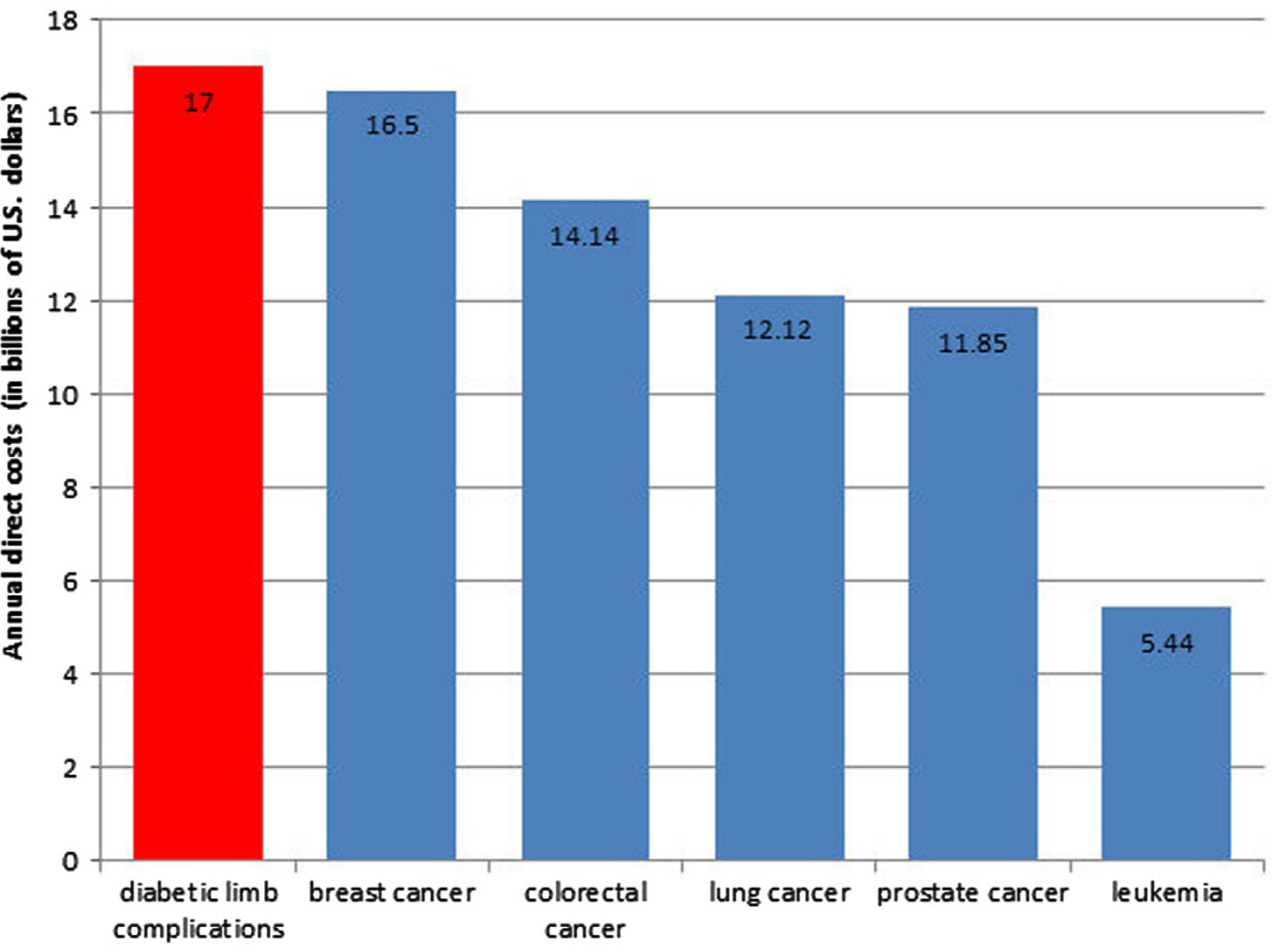
The estimated annual direct costs of diabetic limb complications in comparison to the annual direct costs of the five most costly cancers in the United States.

Diabetic foot care has indeed improved significantly over the past decade. There has been a clearer understanding of the causal factors leading to limb loss and increasing consensus on the management of various aspects of diabetic foot care (15–17). Overall rates of limb loss among people with diabetes appear to be decreasing in the US (18–20) and elsewhere (21–24). At least part of this decrease may be due to improved coordination of care and more frequent interdisciplinary collaboration between specialty providers (21, 22, 25–30). Reform of the US healthcare system through recent legislation (31) designed to improve access to care, encourage more preventative services, and align provider reimbursement with patient-oriented outcomes may provide additional opportunities for US healthcare providers to further improve the system of care for people with diabetes and diabetic limbs.

The current reality in most US healthcare systems is still marked by many significant challenges, however. Limited patient understanding of the potential health significance of a DFU and limited access to care may both have negative impacts (32, 33). Primary care providers perform complete foot examinations only infrequently (34–36) and may lack the time or training (35) to educate at-risk people with diabetes. Adherence to guidelines is uneven (35), and referrals to specialty care can be sporadic (37–39). Beliefs regarding the utility and cost-effectiveness of limb preservation efforts may range from doubt to pessimism and nihilism, even among specialists (40, 41). The resources within any healthcare systems are finite, and the requests for additional providers or funds for quality improvement efforts may be approved only based on the priorities within that healthcare system (35). With these realities and challenges in mind, this review presents a review not only of the patient-related factors but also the provider- and healthcare system-related factors that influence the development of DFUs, the response of DFUs to various treatment modalities, and the progression to limb loss. Through this review, we hope to provide a framework for optimizing patient-oriented outcomes through healthcare system-wide improvements in diabetic foot care in a resource-limited healthcare environment.

## Occurrence and management of an initial DFU

**Fig. 3:**
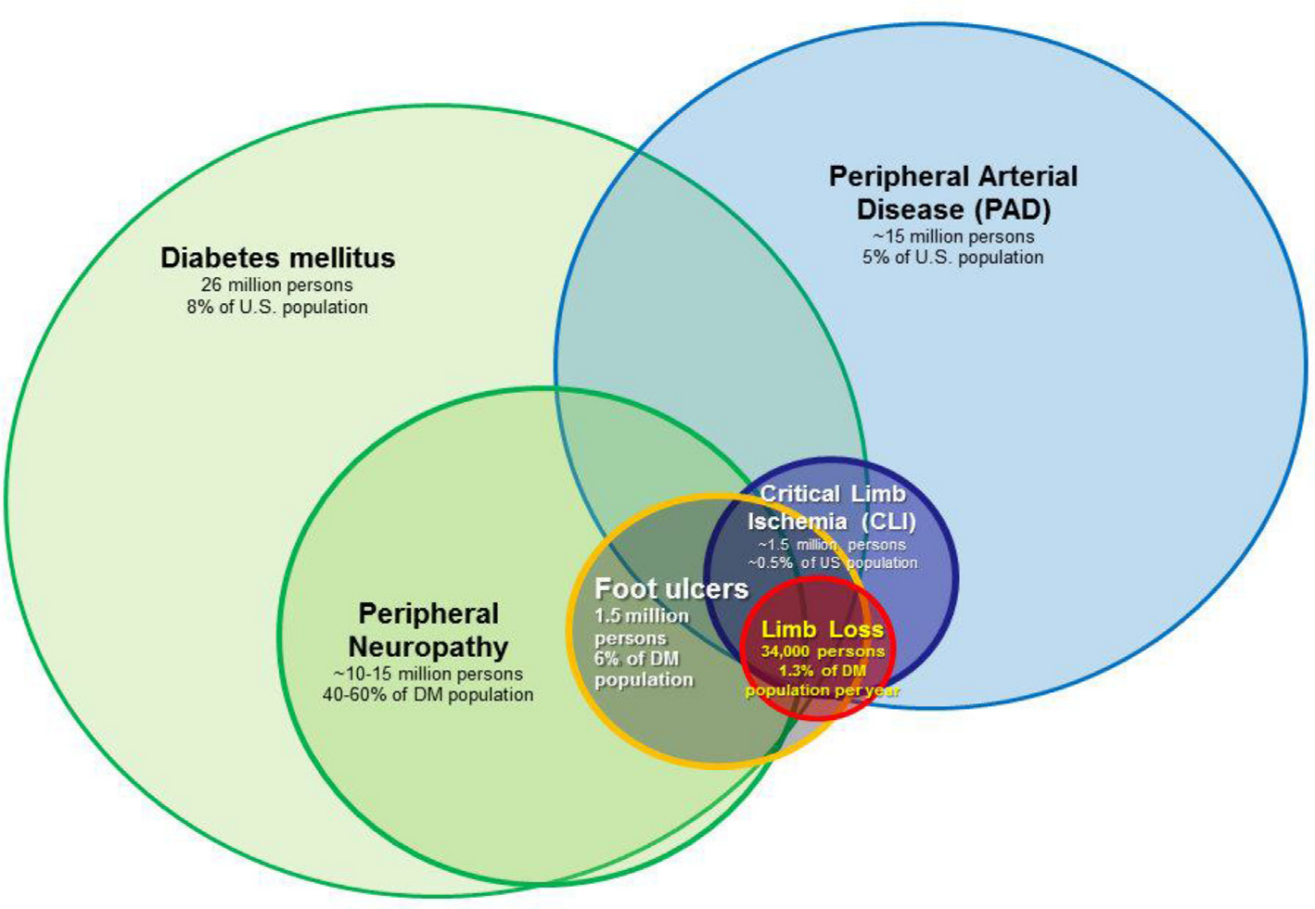
The overlapping relationship of risk factors associated with non-traumatic limb loss in the United States. Estimates of total affected US population, US prevalence and annual incidence rates are shown.

Three factors consistently play an important role in the development of DFUs: structural foot abnormalities, sensory neuropathy, and PAD (Fig. 3). First, the feet of people with diabetes often undergo characteristic structural changes that are the consequence of autonomic and motor neuropathies, intrinsic muscle atrophy, and reduced joint/tendon mobility. Previous minor amputations (i.e. amputations below the level of the ankle, such as toe amputations or partial foot amputations) also result in structural abnormalities (42). The end result of these structural abnormalities is an unequal distribution of stress on the plantar surface of the foot during the gait cycle (43), which in turn predisposes prominent areas of the foot to repetitive trauma and ultimately full-thickness skin ulceration (44). Peripheral sensory neuropathy (also referred to as loss of protective sensation) is a second important factor that leads to DFUs in that it decreases or eliminates the nociceptive response to repetitive trauma that would typically be protective against repetitive trauma occurring during the gait cycle. The prevalence of sensory neuropathy in diabetic populations in the United State and United Kingdom typically ranges between 40 and 60% (45–49) and denotes up to a twofold higher relative risk of DFU incidence (49, 50). The presence of PAD also has a major influence on the development of DFUs (50, 51). The incidence of PAD in the general diabetic population is 20–30%, at least twofold higher than that of non-diabetics. Among those with DFUs, the incidence reaches 50%; (52–54). PAD, diabetes, and peripheral neuropathy are three important risk factors that frequently overlap in patients at risk for limb loss (Fig. 3). Other important risk factors include end-stage renal disease (55, 56), visual impairment (49), improperly fitted shoes (57, 58), autonomic neuropathy (59, 60), and depressive symptoms (61, 62). Poor glycemic control has a well-recognized role in the development of peripheral sensory neuropathy (63) and therefore has at least some causal role in the development of DFUs. It is unclear if glycemic control has a significant impact of limb loss risk independent of the presence or absence of neuropathy, however (64, 65).

DFU treatment is best done through interdisciplinary management (21, 22, 25, 30, 66, 67). Primary care physicians, podiatrists, vascular surgeons, and prosthetists/orthotists typically comprise the core of these teams in most healthcare systems, but wound care clinicians, interventional radiologists and cardiologists, general surgeons, plastic surgeons, orthopedic surgeons, physical therapists, physical medicine and rehabilitation physicians, endocrinologists, and infectious disease specialists may also be involved to varying degrees in many healthcare systems.

Up to 60% of foot ulcers present with some clinical signs of infection (54, 68). The spectrum of infection may include cellulitis, abscess, tenosynovitis, myositis, fasciitis, septic arthritis, osteomyelitis, or some combination thereof (16). Foot infections in patients with diabetes lead to increased healthcare costs and increased risk of limb loss. Thus identifying and treating any infection present is an important initial step in the management of a DFU. Treatment of infection may range from oral antibiotics alone to minor amputations/aggressive foot debridement with wide-spectrum intravenous antibiotics.

Once infection is controlled, further DFU management generally consists of three important components: (i) ensuring/establishing adequate arterial perfusion to the foot; (ii) offloading; and (iii) local wound care (69) (Fig. 1). Evaluating the arterial perfusion to the foot should be the first component undertaken, as offloading and local wound care are unlikely to achieve durable healing if this is not present (54, 70). Revascularization, either in the form of endovascular intervention (i.e. angioplasty) or surgical bypass, increases the likelihood of DFU healing and significantly decreases the risk of limb loss (71). Offloading – reducing pressure in the area of ulceration during the gait cycle – is a second critical component of DFU management (72–75). This may best be achieved by total contact casts or non-removable controlled-ankle movement walkers (43), though surgical procedures [Achilles tendon lengthening (76) and other means (77)] may also be useful in limiting pressure to ulcerated areas of the foot. Diligent local wound care is also important, with debridement done as needed (78, 79). Certain wound care adjuncts – including recombinant platelet-derived growth factors (80–82), negative pressure wound therapy (83–85), and human skin equivalents (86) – may produce improved healing rates compared to standard gauze dressing materials (87). Even with diligent attention to these three components of DFU management, however, healing is often slow [24% healed within 12 weeks (87)] and incomplete [10–20% remaining unhealed at 1 year (88)].

## *DFU primary prevention*: identifying and modifying DFU risk factors to avoid DFU occurrence

Structural foot abnormalities, PAD, and neuropathy are irreversible. Primary prevention efforts have therefore focused on the identification of risk factors, patient education and the promotion of certain health behaviors to minimize foot trauma and avoid delayed presentation. Foot-protective health behaviors often taught to patients with diabetes focus on minimizing the foot trauma through avoidance of barefoot walking, wearing shoes with improper fit, and stepping into bath water without checking the temperature, and monitoring the variability of walking and other activities (89). Between 40 and 90% of patients with neuropathy are unaware of having it (90, 91); these foot protective behaviors might be especially beneficial to these patients who are unaware of their impaired nociception and its potential consequences.

The effectiveness patient education on the prevention of DFUs has been analyzed in a Cochrane database review [most recently updated in 2012 (92)]. This review included 12 randomized clinical trials that assessed the impact of patient education interventions ranging from a 10–20 min educational session to multiple sessions covering various aspects of diabetes management with or without supplementary written materials and/or mailed care reminders for both patients and clinicians. The review concluded that patient education interventions may improve patients’ understanding of foot complications and adherence to certain health behaviors, but there was no consistent evidence to suggest a reduction in the incidence of DFU formation or lower extremity amputation. Only one trial (93) included in the review focused exclusively on primary prevention (i.e. the avoidance of DFU formation among at-risk patients without a previous history of DFU), with the remaining 11 trials including patients with previous DFUs or whose baseline characteristics were not described. A similar review of ‘complex interventions’ (defined as ‘two or more prevention strategies on at least two different levels of care’) also failed to find any clear evidence of benefit in reducing DFU incidence, but the few trials included in the review were small and differed somewhat in the interventions studied.

Mitigating the effects of structural foot abnormalities has been another approach to DFU primary prevention efforts. One randomized clinical trial (94) and at least two non-randomized studies (95, 96) have examined the use of insoles among people with diabetes without a history of previous DFU but who were considered high risk because of pronation, neuropathy, and/or elevated peak plantar pressures. The use of insoles does appear to decrease peak plantar pressures in these studies, but it has not been clearly demonstrated that this translates to a significant reduction in DFU formation.

In addition to the lack of evidence for benefit, there are other obstacles to primary prevention efforts that exist in clinical practice. First, many primary care providers fail to examine the feet of patients with diabetes and infrequently assess for risk factors. In one study of primary care clinic in San Antonio, foot examinations that included an evaluation for neuropathy and PAD occurred in as few as 14% of clinic visits. The foot examination rate was no higher among patients who were known to have PAD, a history of previous foot ulcers, or microvascular complications in other organ systems. A clinic-wide program to improve surveillance for DFUs in this study increased the foot examination rate to only 62% (36). The difficulty of diagnosing PAD in people with diabetes [especially the popliteal and tibial-level distribution most commonly seen in this patient population (97, 98)] may also contribute to this problem. Palpating for pedal pulses does not have good interobserver agreement (99), and even the ankle-brachial indices can be of limited utility in the diagnosis of PAD in the diabetic population (100). Finally, persistent but incorrect beliefs – such as ‘small-vessel disease’ having a causal role in diabetic limb loss (44) – may persist among healthcare providers and lead to a poor understanding of the risk factors involved in DFU formation and limb loss.

The scale of efforts needed for consistent primary prevention efforts is another obstacle. The healthcare system of two of the authors, for example, provides primary care for approximately 110,000 people, approximately 28,000 of whom have diabetes. Even focusing prevention efforts on only moderate- and high-risk individuals would require thorough and accurate risk stratification of all 28,000 patients with diabetes and prevention efforts for approximately 8,000. As in many US healthcare systems, primary care providers at our institution are already challenged to provide basic primary care to patients in a busy outpatient clinic setting; adding additional primary prevention for foot care would simply not be possible without additional personnel and other resources.

## *DFU secondary prevention*: avoiding delays in the recognition of DFUs

Once a DFU has developed, management is best provided by a collaborative team of multidisciplinary specialty providers (21, 22, 25, 30, 66, 67). The provision of this multidisciplinary care is predicated on both the identification of a DFU and access to medical care. Primary care providers and specialty providers (especially podiatrists, vascular surgeons, and endocrinologists) may identify DFUs not previously noted or treated, patients themselves are the most important persons who can identify a new DFU and seek treatment for it. Many patient-related barriers to prompt recognition of DFUs exist, however. One study of veterans with diabetes found that only 32% examined their feet on a daily basis (101). A daily self-foot examination is not likely to be done or be helpful if patients have not been instructed to do these examinations or if the patient does not known what an ‘ulcer’ or other foot abnormalities looks like (33). Visual impairment, poor balance/equilibrium, decreased limb flexibility, and obesity may also limit a patient's ability to examine the plantar aspect of his or her foot and recognize the abnormalities. Even when abnormalities are found, patients may not immediately seek medical attention because they are unaware of the relationship between DFUs and limb loss, have limited access to medical care (102), or do not know what type of provider to see (32). Even after adjusting for DFU incidence, demographics, and other important variables, US patients with lower socioeconomic status have a higher risk of limb loss (103), and this does not appear to be related to the prevalence of physicians and/or podiatrists in a given area (103).

Patients with recognized DFUs should generally be referred for multidisciplinary specialist foot care, but these referrals are frequently delayed or absent. In the EuroDIALE study, for example, 27% of the patients were referred to a specialist foot clinic only after the DFU had been present for more than 3 months. Specialist referrals varied widely not only between countries but also among centers within a country (39). Such delays can negatively impact outcomes (37). Access to the specialist clinic may remain a problem even after a referral is made, as long delays between referral and appointments are common. The creation of open access facilities – i.e. clinics or centers where ‘walk in’ appointments or urgent referrals are seen whenever needed – may be one method to avoid delays in treatment and significantly improve clinical outcomes (104).

**Fig. 4:**
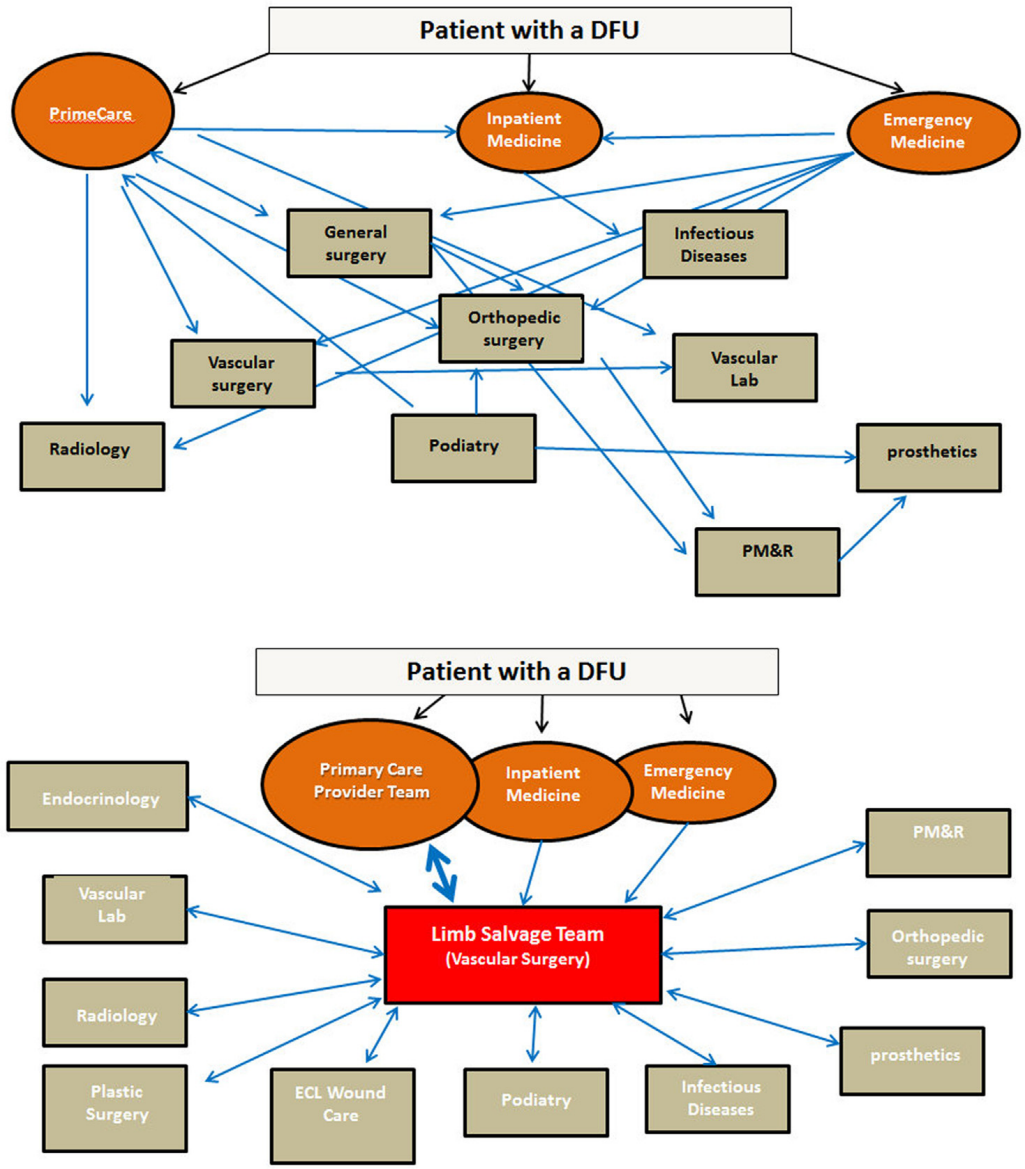
Schematic diagram demonstrating (A) an example of a disordered, *ad hoc* pattern of patient referral and communication in among coalition of relevant specialty providers; and (B) an example of more structured patient referral and communication in a multidisciplinary care team.

The wide array of specialist providers involved in various aspects of DFU management may introduce some confusion into the referral process. The relationship between the various specialist providers involved in any given patient's care may range from an *ad hoc* collection of specialists with no or infrequent communication within some healthcare systems to a formalized team with robust, regular communication in others. The providers may be located in separate clinics scattered across a geographical area, located in separate clinics of a large hospital, or physically co-located in one clinic. Following from this, the referral of patients with DFUs to particular specialists may range from uncertain, sporadic and determined on an individual basis by the primary provider the patient happens to be seeing to a consistent multidisciplinary team with a formalized referral protocol (Fig. 4).

## *DFU tertiary prevention*: ensuring adequate DFU treatment

Other barriers to optimal care persist even after a patient with a DFU is seen by a specialist. Perhaps the most important is specialty referral or practice heterogeneity. Only 41% of patients with PAD in the EuroDIALE study received vascular imaging, and only 43% of patients with severe limb ischemia underwent revascularization (39). The possibility that such practice heterogeneity affects major amputation rates has been suggested by multiple studies of US administrative data demonstrating decreased revascularization among blacks with DFUs (105–107). Some of the variation in amputation rates is due to variations in patient presentation (108), and geographic variations in amputation rates are seen even in nationalized healthcare systems in the US (109) and the UK (110). Regardless, the findings of the Medicare studies and others (103, 111, 112) do suggest practice heterogeneity may have at least some negative impact on patient outcomes.

Additional provider-related factors influence diabetic foot outcomes. Providers may not be aware of national or international consensus treatment guidelines, may not have established local consensus guidelines (35), or may have differing opinions on the proper treatment for an individual patient (113, 114). Patients who undergo revascularization at low volume medical centers may have a small but significant increase in the risk of limb loss and mortality compared to those who undergo revascularization at high volume centers (115). US President Barack Obama had suggested that the higher relative monetary reimbursement for major amputation versus revascularization may lead to a higher propensity to perform amputation (116). The relative reimbursement amount as measured by relative value units per median procedural times for infrainguinal revascularization is indeed much lower than that of major amputations (117), and although there are no data to suggest that this differential reimbursement rate does indeed affect propensity to pursue limb preservation efforts over major amputation, many others have suggested the need to better align provider reimbursement with the provision of patient-oriented outcomes (118).

## Reducing ulcer recurrence

In large observational series of patients with DFUs, the risk of DFU recurrence reaches 40–60% (119, 120). Two factors seem to be largely responsible for this high recurrence rate. First, a healed DFU remains at increased risk because of abnormal epidermis/dermis structure. Second, DFUs most often occur in patients with known risk factors; although the DFU may heal, these risk factors typically remain.

Studies of patient education interventions to prevent DFU recurrence have reported somewhat contradictory results (120, 121), but as mentioned above there is overall no clear evidence that patient education has a significant impact on reducing DFU recurrence (92). The continued use of modified therapeutic shoes appears to have a significant impact on reducing DFU recurrence compared to normal shoes (28% vs. 58% recurrence at 1 year, respectively) (122). Daily thermography has been demonstrated to significantly reduce reulceration rates. With daily thermography, patients plantar foot skin temperature gradients by standing on a thermography scale or using a specialized cutaneous temperature probe. If a gradient of ≥5°F is found, walking is minimized until the foot temperatures equilibrate. Three randomized trials in 483 patients with a history of previous DFU have demonstrated rather impressive reductions: from 6-month DFU incidence rates of 20% with standard care to 2% with daily thermography (123–125).

## Conclusions: prioritizing quality improvement efforts for diabetic foot care

Optimal management of the diabetic foot for the minimization of limb loss truly requires a robust system of multidisciplinary care (Table 2). This system of care is not just a single provider or specialty but an array of providers, ideally working in a cohesive, multidisciplinary team; it is not simply management of the DFU but also screening, education, and surveillance; and it should target not only those referred for care but also patients not currently receiving care but who are in need of it. So can such robust systems of care be established? It can be challenging even within nationalized healthcare systems (35); it can be even more challenging where healthcare systems are composed of individuals or groups that each have a selective area of focus and lack incentive (financial or otherwise) to organize and provide care across the full spectrum of disease.

**Table 2:** Components of diabetic foot care and respective objectives

Component		Priorities
Primary prevention	Avoiding DFU occurrence	Identifying moderate- and high-risk patients with diabetes Establishing the impact of primary prevention efforts
Secondary prevention	Promptly identifying DFUs and accessing care	Increase access to primary and/or specialist care (open access, other)
Tertiary prevention (i.e. DFU management)	Ensuring adequate DFU care to minimize the risk of limb loss	Consistent management algorithms to Ensuring/establishing adequate arterial perfusion to the foot Mechanical offloading Local wound care
Reducing recurrence	Avoiding DFU recurrence	Providing long-term options for offloading the at-risk area

DFU, diabetic foot ulcer.

First, the role of primary prevention needs to be clarified. Few randomized trials have examined the impact of primary prevention efforts. Larger, better quality trials – perhaps with accompanying economic analyses – may be needed to more convincingly demonstrate the benefit of various forms of primary prevention. Recurrence of DFU is frequent, and better methods for reducing this recurrence rate are needed (126).

Delays in the recognition of PAD appear to be common. Given the impact of PAD on DFU outcomes, efforts to reduce delays in the recognition and treatment of PAD among patients with DFUs are well deserved (2). In spite of the higher initial costs associated with treatment, limb preservation efforts appear to be cost-effective and, in some situations, may provide cost-savings (127). The number of publications from healthcare systems reporting significant decreases in major amputations rates continues to grow (21, 22, 25, 30, 67), but still the existence of robust systems of multidisciplinary care is somewhat sporadic in the United States. Quantitative studies that assess not only the impact of various prevention and treatment strategies but also the impact of delays might help provide a further (esp. economic) argument for establishing a robust system of diabetic foot care.

Finally, while the economic, functional and psychosocial impacts of diabetic foot complications are difficult to overstate, there exists a yawning gap between the impact of this problem and funding for research to improve management. Of more than 22,000 diabetes-related research projects with US federal funding between 2002 and 2011, only 33 (0.15%) were related to DFU care. Although diabetic foot complications may comprise as much as 30% of the excess medical costs of patients with diabetes (128), the cumulative funding for these projects accounted for only 0.17% of the total US funding for diabetes-related research, a >600-fold difference (129). More support through improved federal funding for DFU-related research may help produce meaningful therapeutic improvements in this area.
